# Brain imaging and morphological plasticity in glioblastoma: a literature review

**DOI:** 10.25122/jml-2022-0201

**Published:** 2023-03

**Authors:** Andreea-Anamaria Idu, Nicolae-Stefan Bogaciu, Alexandru Vlad Ciurea

**Affiliations:** 1 Department of Neurosurgery, Henri Mondor Hospital, Créteil, France; 2 Department of Neurosurgery, Annecy-Genevois Hospital, Annecy, France; 3 Department of Neurosurgery, Sanador Hospital, Bucharest, Romania; 4 Clinical Neurosciences Department, Carol Davila University of Medicine and Pharmacy, Bucharest, Romania

**Keywords:** glioblastoma, functional MRI, neuroplasticity

## Abstract

This article provides a comprehensive review of the role of functional magnetic resonance imaging (fMRI) in characterizing neural plasticity in glioblastoma patients. Glioblastoma, the most common primary brain tumor, has a rapid growth rate and infiltrative nature that leads to the disorganization of the normal brain network. Neuroplasticity, still not fully understood, is the foundation for the development of brain functions during the growth and recovery of certain brain functions after a brain lesion such as a tumor, trauma, or vascular event. Functional MRI has the capacity to identify the regions that activate at rest or when performing a task. It can determine the extent to which these regions, responsible for a specific function, are impacted by a tumor and eventually after surgical excision. Likewise, it can help evaluate to which extent activation changes when recovery of function occurs. In this article, we aimed to understand the significance of fMRI in the management of glioblastoma by analyzing representative articles from the literature.

## INTRODUCTION

Glioblastoma is the most frequent primary central nervous system tumor, characterized by its aggressive nature and a very poor prognosis. Patients diagnosed with this tumor are usually over the age of 40 (in the 65-75 interval) and have a male predominance of 1.5 :1 [1]. Magnetic resonance imaging (MRI) of glioblastoma shows hypo to iso-intensity on T1 images and hyperintensity on T2 and FLAIR sequences, with observable perilesional edema. DWI/ADC sequences show a restriction diffusion of the solid component [1,2].

Functional MRI (fMRI) allows for the identification of brain areas under specific stimulation through the BOLD (blood oxygen level dependent) mechanism. Essentially, fMRI (based on a T2* sequence) highlights areas with increased cellular activity based on increased oxygen supply, serving as indirect evidence of neuronal activity. Besides its usefulness, fMRI has several disadvantages, including heterogeneity of the field, particularly at the interface between tissues (bone, soft tissue, and air), interference with blood flow in large veins, and metallic artifacts [3,4].

Neuroplasticity refers to the brain's ability to adapt its structural and functional architecture through specific processes in response to external stressors. This adaptation is facilitated by the network-like organization of the central nervous system, with neurons connected by edges. Additionally, neuroplasticity includes the regeneration of neurons in specific areas, such as the dentate gyrus of the hippocampus and the lateral subventricular area [5-8].

Several techniques have been developed to investigate the structural and functional aspects of the brain, including electroencephalography, magnetoencephalography, imaging techniques (computed tomography, positron emission tomography-computed tomography, MRI, fMRI, diffusion tensor imaging), and direct cortical stimulation (considered the gold standard for functional exploration of the brain) [6].

William R. Gibb *et al*. used direct cortical stimulation to observe changes in function after stimulation of the same cortical area in two different surgeries as a way to highlight neuroplasticity. Changes in function can include gains, losses, or alterations, and unexpected responses in the presence of a tumor also indicate neuroplasticity [9]. In fMRI, plasticity is defined as differences in cortical activation during the same task pre-and post-operative imaging. Furthermore, the activation of an unexpected region in the setting of a tumor provides evidence for neuroplasticity [9, 10].

The goal of this review was to explore how imaging techniques, specifically functional MRI, can objectively highlight neuroplasticity and to determine if these techniques have any clinical applications.

## MATERIAL AND METHODS

We conducted a comprehensive search on PubMed using the keywords "glioblastoma," "plasticity," and "MRI." Our search resulted in 33 articles that were relevant to our topic of interest. After thoroughly reviewing the titles and abstracts of these articles, we identified 6 articles that were of particular interest to our research. By following a snowball effect, we were able to locate an additional article that was relevant to our subject. A summary of these articles can be found in Table 1.

**Table 1:** Studies included in the literature review - publication titles, authors, year of publication, and number of patients included.

Title	Authors	Year	No. patients
Direct Evidence of Plasticity within Human Primary Motor and Somatosensory Cortices of Patients with Glioblastoma	William R. Gibb, Nathan W. Kong, Matthew C. Tate	2020	6
Functional MRI Studies of the Hemisphere Dominant for Speech in Patients with Brain Tumors	S.B. Buklina, A.E. Podoprigora, I.N. Pronin, L.V. Shishkina, G.N. Boldireva, A.A. Bondarenko, L.M. Fadeeva, V.N. Kornienko, V.Yu. Zhukov	2013	21
Precentral Glioma Location Determines the Displacement of Cortical Hand Representation	Gilbert Wunderlich, Uwe Knorr, Hans Herzog, Jurgen C.W. Kiwit, Hans-Joachim Freund, Rudiger J. Seitz	1998	6
Plastic reshaping of cortical language areas evaluated by navigated transcranial magnetic stimulation in a surgical case of glioblastoma multiforme	Akitsugu Kawashimaa, Sandro M. Krieg, Katharina Faust, Heike Schneider, Peter Vajkoczyc, Thomas Picht	2013	1
Connectome analysis for pre-operative brain mapping in neurosurgery	Michael G. Hart, Stephen J. Price & John Suckling	2016	5
“I do not feel my hand where I see it”: causal mapping of visuo-proprioceptive integration network in a surgical glioma patient	Emmanuel Mandonnet, Daniel Margulies, Chloe Stenge, Mélissa Dali, François Rheault, Monica N. Toba,François Bonnetblanc, Antoni Valero-Cabre	2020	1
Process of the Functional Reorganization of the Cortical Centers for Movement in GBM Patients: fMRI Study	A. Majos, B. Bryszewski, KN. Kośla, L. Pfaifer, D. Jaskólski, L. Stefańczyk,	2015	16

## RESULTS

A summary of all glioblastoma cases with different plasticity assessment techniques and interpretation of results is presented in Table 2. However, it can be noted that there are not many articles in the literature concerning neuroplasticity in the context of glioblastoma. The articles that do exist often refer to low-grade gliomas, which have a slower and longer evolution than glioblastomas. Despite these limitations, we were able to identify several articles that also discuss high-grade tumors. These articles cover case reports of glioblastomas included in heterogeneous series, as well as homogeneous, uni-pathological series. Various techniques have been employed to evaluate changes in brain function in different regions, including imaging techniques, non-invasive stimulation/recording techniques, and invasive stimulation/recording techniques. Of all these techniques, direct cortical stimulation is considered the gold standard.

**Table 2:** Synthesis of glioblastoma cases found in the studies. It should be noted that in most cases, neuroplasticity has been identified by various techniques. As for functional MRI, there is lower reliability than DES (direct cortical stimulation) which is the standard for functional evaluation.

No	Author	No patients	Technique	Result interpretation
1	Wunderlich *et al*.	1	fMRI + PET scan + TMS	Activation of the fronto-mesial cortex, including the representation of the lower limb and the supplementary motor area - for the movement of the hand
2	Buklina *et al*.	7	fMRI	Search for Broca’s area – no activation on fMRI in studied cases
3	Kawashima *et al*.	1	nrTMS	Activation of the contralateral hemisphere after surgical resection of glioblastoma and activation of post-central and temporal areas not previously activated
4	Mandonnet *et al*.	1	DES, fMRI evoked potentials	Activation of the contralateral hemisphere in the setting of a left superior parietal lobule lesion, confirmed by DES and also described on fMRI
5	Gibb *et al*.	6	DES	Evidence of plasticity in all cases
6	Majos *et al*.	16	fMRI	Activation of the supplementary motor area (12 cases) and pre-motor area (8 cases) contralateral to the lesion as visualized on fMRI

In the article by Wunderlich *et al*. from 1998, only one out of 6 studied cases was diagnosed with glioblastoma. The inclusion criteria for the study were precentral gyrus tumors found on MRI without other suspect lesions. All patients underwent MRI and PET-CT, and transcranial magnetic stimulation was performed to register activity in the first interosseous muscle. Notably, despite an increase in the regional cerebral blood flow (rCBF) in the respective pre-central gyrus and a displacement of the central sulcus in all other cases, the glioblastoma patient did not exhibit any of these characteristics [11].

Buklina *et al*. (2013) evaluated the dominant hemisphere using functional MRI for language area assessment in 21 patients, seven of whom were diagnosed with glioblastoma. They found that activation of language areas varied significantly depending on the tests performed, both among different patients and in the same patient. However, the study failed to identify Broca’s area in any of the glioblastoma patients [12].

Identifying activation zones for certain actions can be challenging in the presence of a large tumor, particularly a high-grade glioblastoma. Functional MRI depends on the specific signal of BOLD to identify the activation zones when action is exerted. This signal can be distorted by the presence of neovascularization in the tumor, hindered arterial and venous blood flow caused by edema, and mass effect around the affected hemisphere [12].

Kawashima *et al*. (2013) presented a case of glioblastoma in which they highlighted the reorganization process of the language cortical areas using a recent procedure called navigated repetitive transcranial magnetic stimulation (nrTMS). While functional MRI did not contribute to this case, the conclusion underlines the need for non-invasive evaluation of neuroplasticity in the setting of a fast-evolving tumor like glioblastoma [13].

To understand neuronal connectivity and its dynamism in the context of a brain tumor, Hart *et al*. performed a connectome analysis on 5 patients with glioblastoma. This study attempted to create a model of the functional neural network based on the acquisition of resting functional MRI data for this disease. Graph theory was utilized to create network models with nodes and edges based on the statistical analysis of probabilities. The models were used to make predictions about the presence of the brain tumor. These predictions include both the effect of the tumor on the connectome, but also the effect of surgical excision of the tumors on the connectivity. They identified that certain nodes and edges were disproportionately more vulnerable than others. The study demonstrated that the presence of glioblastomas has a rapid and aggressive evolution that creates connectivity problems at all levels: lobar, whole hemisphere, and inter-hemispheric. These findings demonstrate the potential of functional MRI in realizing the connectome of the brain of a patient with glioblastoma, which could improve the extent of the resection in preoperative planning [14].

Majos *et al*. (2015) examined 16 patients with grade IV glial lesions in the central sulcus using preoperative functional MRI. Only 9 patients underwent a second evaluation at 3 months postoperatively. They observed that the secondary movement control centers of the counter-lateral hemisphere were the first to activate in patients with tumors smaller than 40cm3, notably the supplementary motor area and the promoter cortex. The ipsilateral centers, such as the premotor and motor areas, respectively, were less frequently activated. Motor area activation on both sides was less evident in the larger tumor cases. Despite observing reorganization in other areas, there was no change in activity in the contralateral supplementary motor area and a less obvious change in the contralateral premotor area on the second functional imaging three months after the intervention. However, it is important to note that there was still neurological deterioration. Therefore, the authors concluded that the contralateral supplementary motor area played a primary role in reorganization in the context of a high-grade glial lesion, while the contralateral premotor area played a secondary role [15].

In a case report by Mandonnet *et al*. (2020), a patient with a glioblastoma in the left superior parietal lobule exhibited visuospatial integration disturbance and reported a mismatch between the position seen and the position perceived of her right upper limb during stimulation of the affected area. Connectivity analysis using resting functional MRI showed that the node responsible for integrating visuospatial perception could be relocated from side to side in the context of a brain tumor at that location. The preservation of interhemispheric commissural fibers suggests that mechanisms of neuronal plasticity worked in this case [16].

The study by S.B. Buklina highlights the lack of reliability of functional MRI in obtaining useful recordings for patients with glioblastomas. The authors attribute this problem to neurovascular decoupling caused by the tumor, which is more pronounced in high-grade gliomas with accelerated growth and greater mass effect that leads to changes in the cerebral circulation. To overcome this limitation of the BOLD technique, alternative methods such as molecular markers of neuronal cell activity, as studied using spectroscopy sequences, or other new techniques adapted for use in the human brain, such as those used on insects or small fish, may be considered. Nonetheless, significant interest remains in the non-invasive evaluation of brain activity, particularly in the presence of brain tumors [17,18]. Some authors have found satisfactory results with navigated repetitive transcranial magnetic stimulation (nrTMS) technique. In comparison with the direct cortical stimulation technique, the results were well correlated in the article by Motomura *et al*. [19].

Identifying the cerebral areas involved in different functions is crucial in glioma surgery, particularly in achieving "maximum safe resection" or " maximum resection under safe conditions”. This means removing as much of the tumor as possible without creating new neurological disorders, which is directly correlated with survival and recurrence rates. In the context of high-grade gliomas, which have a faster rate of progression and growth, the objective of achieving maximum safe resection becomes even more crucial [20].

Functional MRI is a widely available and non-invasive technique that can be performed on any 3 or even 1.5-Tesla device, making it a more accessible option than invasive stimulation techniques such as direct cortical stimulation. It provides anatomical images with good resolution (more easily recognizable by a neurosurgeon). However, as discussed earlier, there are also disadvantages of functional MRI [21].

After reviewing the literature, we have found that functional MRI is a valuable tool for providing information about brain function and connectivity. This technique has been used to map the connectivity of the brain and predict changes in function in certain nodes of the neuronal network. Anatomically, functional imaging has allowed us to observe the different displacements of function and identify certain activation patterns in several patients. The plasticity of the brain has been proven by functional imaging in the majority of the reviewed articles, demonstrating its usefulness and effectiveness.

Functional imaging is particularly useful in the preoperative planning for glioblastoma resection surgery, although it cannot be the sole basis for a surgical decision. Follow-up using functional imaging is also beneficial for patients with glioblastomas. This approach facilitates a comparison of changes in areas of activation over time, including a preoperative baseline, immediate postoperative assessment within 48-72 hours, and later time points. As direct cortical stimulation is not feasible at each follow-up, functional imaging presents a useful tool in this case.

## CONCLUSION

In this study, we conducted a bibliographic research on functional imaging in the context of glioblastoma diagnosis. Our findings suggest that functional MRI is a readily available tool for evaluating brain functionality that could be systematically included in the preoperative planning of glioblastomas.

One of the most significant advantages of functional MRI is its non-invasive feature, which is accompanied by other benefits, such as the wide availability, production of anatomical images, and the ability to repeat the examination without causing harm. In addition, the use of follow-up functional MRI to assess the evolution of cerebral plasticity is an interesting possibility. However, there are still some drawbacks to using functional MRI, such as neurovascular decoupling and the lack of reliability in making surgical decisions. In conclusion, we believe that functional MRI has great potential and can open doors to new techniques in the diagnosis and management of glioblastomas. Our study highlights the importance of this non-invasive tool and suggests that it can be beneficial in clinical settings.
